# The complete chloroplast genome sequence of *Styrax duclouxii* Perkins (Styracaceae)

**DOI:** 10.1080/23802359.2020.1722039

**Published:** 2020-02-03

**Authors:** Xiaogang Xu, Yaoqin Zhang, Lili Tong, Yabo Wang, Chongli Xia

**Affiliations:** a Co-Innovation Center for Sustainable Forestry in Southern China, College of Biology and the Environment, Key Laboratory of State Forestry and Grassland Administration on Subtropical Forest Biodiversity Conservation, Nanjing Forestry University, Nanjing, China;; b State Environmental Protection Scientific Observation and Research Station for Ecology and Environment of Wuyi Mountains, Nanping, China;; c School of Horticulture & Landscape Architecture, Jinling Institute of Technology, Nanjing, China

**Keywords:** *Styrax duclouxii*, Styracaceae, complete chloroplast genome, phylogenetic analysis

## Abstract

The object of this work was to measure the complete chloroplast genome of *Styrax duclouxii* Perkins for the sake of offering valuable genomic information to promote its conservation. The circular genome of *S. duclouxii* was measured as 157,913 bp in size and contained two inverted repeat (IRa and IRb) regions of 26,040 bp, which were separated by a large single-copy (LSC) region of 87,604 bp and a small single copy (SSC) region of 18,229 bp. A total of 134 genes are encoded, including 89 protein-coding genes, 37 tRNA genes, and 8 rRNA genes. The overall GC content of *Schizosaccharomyces japonicus* genome is 36.97%. A phylogenetic tree reconstructed using 36 chloroplast genomes reveals that *S. duclouxii* is most closely related with *Styrax zhejiangensis* and *Styrax faberi.*

*Styrax duclouxii* Perkins is widely distributed across China, Korea, and Japan, which has medicinal value, aromatic property, ornamental, and timber resources. In this work, we characterized the complete cp genome sequence of *S. duclouxii* (GeneBank accession number: MN882545) based on the date of genome sequencing.

The total genomic DNA was extracted from the fresh leaves of *S. duclouxii* grown in Nanjing Forestry University campus (N 32.0803, E 118.6066) in Nanjing, Jiangsu, China. The voucher specimen was kept in the herbarium of Nanjing Forestry University (accession number: NF2019128). The whole genome sequencing was conducted by Nanjing Genepioneer Biotechnologies Inc. (Nanjing, China) on the Illumina Hiseq platform. The original reads were filtered by CLC Genomics Workbench v9, and the obtained clean reads were assembled into chloroplast genome using SPAdes assembler v3.10.1 (Bankevich et al. 2012). Finally, gene structure annotation was carried out with CpGAVAS (Liu et al. 2012), and the sequences were aligned by MAFFT v7.307 (Katoh and Standley 2014). A neighbor-joining (NJ) tree with 100 bootstrap replicates was inferred using MEGA version 7 (Kumar et al. 2016).

The circular genome of *S. duclouxii* was 157,913 bp in size and contained two inverted repeat (IRa and IRb) regions of 26,040 bp, which were separated by a large single-copy (LSC) region of 87,604 bp and a small single-copy (SSC) region of 18,229 bp. A total of 134 genes are encoded, including 89 protein-coding genes (81 PCG species), 37 tRNA genes (30 tRNA species), and 8 rRNA genes (four rRNA species). Most of the genes occurred in a single copy; however, 8 protein-coding genes (*ndhB, rpl2, rpl23, rps12, rps7, ycf1, ycf15* and *ycf2*), 7 tRNA genes (*trnA-UGC, trnI-CAU, trnI-GAU, trnL-CAA, trnN-GUU, trnR-ACG,* and *trnV-GAC)*, and 4 rRNA genes (*16S, 23S, 4.5S,* and *5S*) are totally duplicated. A total of 9 protein-coding genes (*atpF, ndhA, ndhB, petB, petD, rpl16, rpl2, rpoC1,* and *rps16*) contained 1 intron while the other 3 genes (*clpP, rps12, and ycf3*) had 2 intron each. The overall GC content of *S. duclouxii* genome is 36.97%, and the corresponding values in LSC, SSC, and IR regions are 34.81%, 30.30%, and 42.95%, respectively.

To ascertain the phylogenetic evolution of *S. duclouxii*, the fasta format file containing all the chloroplast genome sequences of 36 species was used(31 Styracaceae chloroplast genomes, 2 Symplocaceae chloroplast genomes, 1 Clethraceae chloroplast genomes, and 2 Actinidiaceae chloroplast genomestaxa). The phylogenetic analysis suggests that *S. duclouxii* is a sister species to *Styrax zhejiangensis* and *Styrax faberi* in Styracaceae, with bootstrap support values of 100% and 87%, respectively (Figure 1).

**Figure 1.**
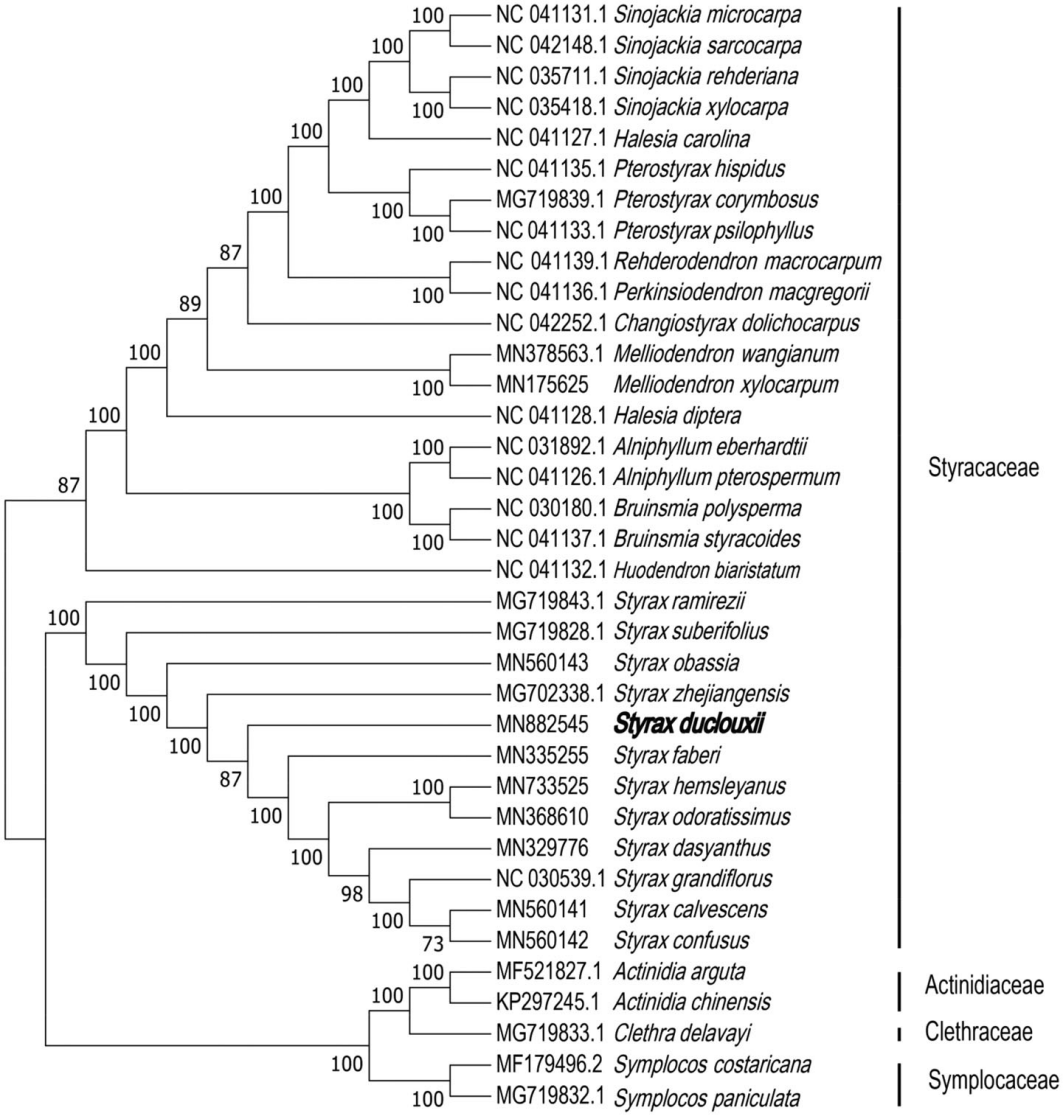
Phylogenetic tree inferred by neighbor-joining (NJ) method based on the complete chloroplast genome of 36 representative species. The bootstrap support values are shown at the branches.
